# Dimensions and position of the Eustachian tube in Humans

**DOI:** 10.1371/journal.pone.0232655

**Published:** 2020-05-04

**Authors:** Ida Janzen-Senn, Robert A. Schuon, Frank Tavassol, Thomas Lenarz, Gerrit Paasche

**Affiliations:** 1 Department of Otolaryngology, Hannover Medical School, Hannover, Germany; 2 Hannover Medical School, Clinic for Cranio-Maxillo-Facial Surgery, Hannover, Germany; 3 Hannover Medical School, Hearing4all Cluster of Excellence, Hannover, Germany; University of Porto Faculty of Medicine, PORTUGAL

## Abstract

Eustachian tube (ET) dysfunction is one of the causes for chronic otitis media. To develop new therapies such as stents to facilitate middle ear ventilation, a better knowledge on dimensions and positions of the ET in individual patients is necessary. Cone beam CT scans of 143 patients were retrospectively investigated. Parameters such as lengths of the ET and its cartilaginous and bony parts, diameters, angles as well as distance of the ostium from the nasal conchae were determined and evaluated for side, gender and age specific differences. The average length of the cartilaginous and bony tubes was smaller in women than men. The average deviation from the horizontal plane was 1.7° larger on the left side (35.4°) compared to the right side (33.7°). Tools to manipulate the ET or to insert stents into the ET should cover angles from at least 42° to 64°. The distance of the pharyngeal orifices from the conchae nasalis inferior increased with age, becoming most prominent above 70 years of age. This investigation provides necessary information to develop stents for human application and tools for safe positioning of the stents.

## Introduction

Otitis media still is one of the most common children’s diseases and the most frequent cause to seek medical advice [1]. The cause of an otitis media often is not completely clear [2], but a dysfunctional Eustachian tube contributes to middle ear effusion and alteration of the mucous membrane [3] which can also lead to retraction and/or perforation of the tympanic membrane [2] or contribute to the formation of cholesteatoma [4].

The Eustachian tube (ET), also called auditory tube or pharyngotympanic tube, is part of a system comprising nose, palate, nasopharynx and middle ear [5]. It is lined with epithelial cells, forms the only connection between middle ear and nasopharynx [6], and its main functions are ventilation of the middle ear, clearance of secretion, and protection against direct sound transmission and pathogenic microorganisms [7]. ET dysfunction, which has an incidence of about 1% for adults [8], can be connected to a number of different symptoms but nevertheless is still not clearly defined [9].

First therapeutic approaches to treat ET dysfunction are Valsalva-maneuver, nasal douche with sodium chloride solution or nasal sprays containing decongestants or corticoids. More invasive treatment options are paracentesis of the tympanic membrane if necessary with the introduction of a grommet [2], laser tuboplasty [2,10] and more recently balloon dilatation of the Eustachian tube (BET) [11,12]. All these treatment options can provide benefits, but the success rate of most of them especially if seen on the long term remains under debate. A detailed review on possible treatments comes to the conclusion that no trial on any of these interventions can currently be recommended and that a clear definition of ET dysfunction in adults is still missing [13].

Additionally, there are new treatment options–such as stents for the ET–under development striving to facilitate ventilation of the middle ear and enable clearance of secretion. These stents shall be inserted into the ET via nose and pharyngeal orifice of the ET and underwent first successful tests in cadavers [14] and sheep [15]. In order to ensure safe application, stent sizes have to be appropriate for the individual patient and tools for atraumatic positioning have to be developed.

Therefore, the aim of the current study was to evaluate the dimensions and position of the human ET and to determine space limitations for possible insertion instruments based on a retrospective evaluation of clinically available cone beam CT scans with special emphasis to possible variations with side, gender, and age.

## Material and methods

### Patients

**Fig 1:**
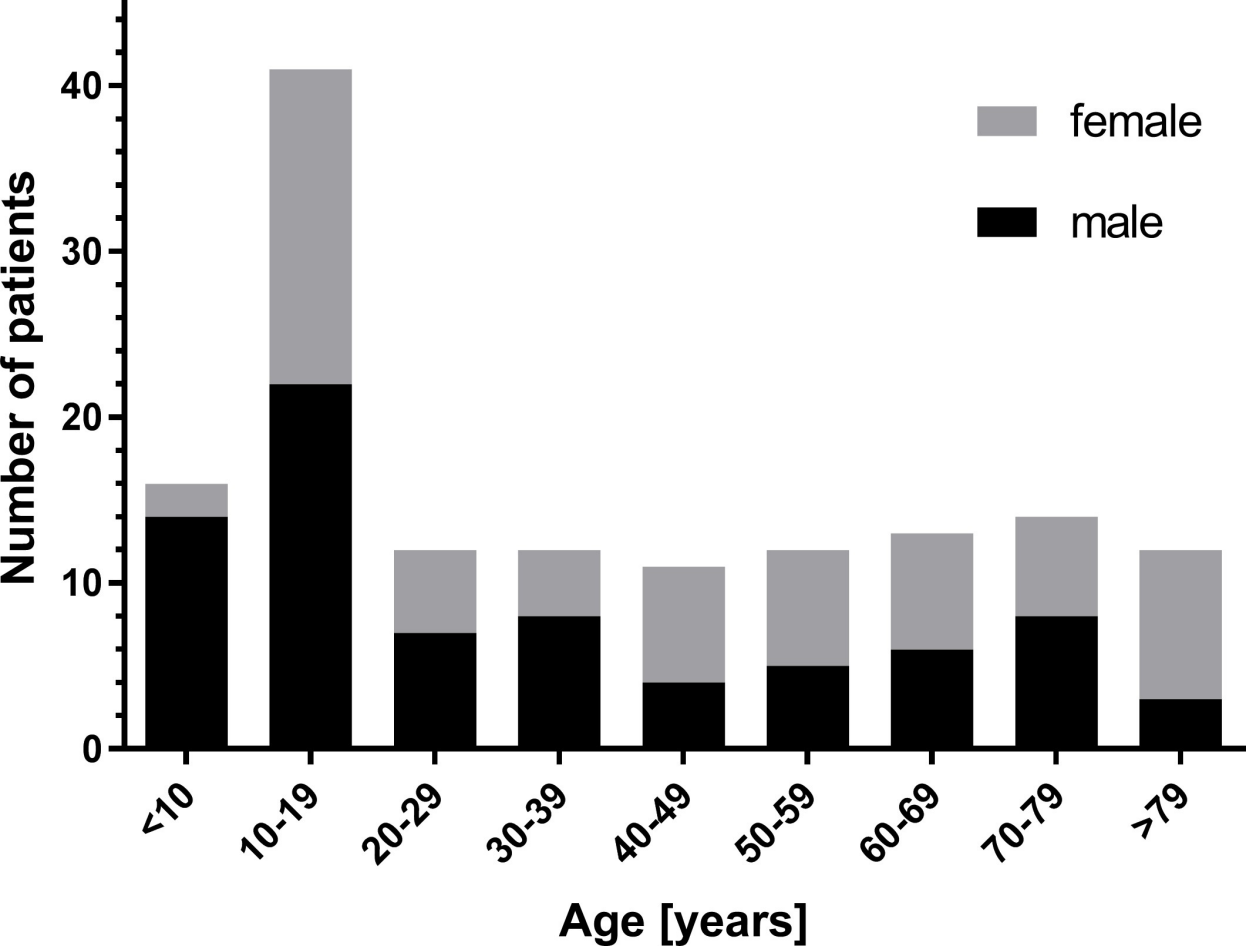
Age distribution of all investigated 143 patients.

Patients undergoing cone beam computer tomography (CBCT) in the clinic for Cranio-Maxillo-Facial Surgery between 2012 and 2017 for different reasons such as exclusion of fracture after trauma and preoperative planning of surgeries for dysgnathia or insertion of dental implants were retrospectively evaluated. Included were only patients with the complete ET, palate and nasopharynx on the scans. Amongst the 143 included patients (bilateral analysis: 286 ET’s) were 77 males and 66 females between 4 and 94 years with 96 of them being 18 years of age or older. Age and gender distributions are provided in Fig 1.

Patient folders were evaluated regarding any pre-existing conditions or interventions that might affect the anatomy of the ET, nasopharynx or middle ear. Due to orofacial cleft, dysgnathia, other anomalies or a tumor in the nasopharynx, 14 patients were excluded from statistical evaluation and additional 2 patients because of poor image quality. All patients or their parents agreed to their data being used for scientific studies and provided written informed consent before performing the respective diagnostics. Ethics Committee of Hannover Medical School written approval was granted under number 3623–2017 before the start of the study.

### CBCT

Scans were performed at 105 kV and 5.1 mA using a PaX-Zenith3D system (VA TECH, Hwaseong, Korea). The field of view was defined at 160x140 mm with a resolution of 0.3 mm in all 3 dimensions. Window width and window level were set between 4200 and 9500, and 1000 and 2500, respectively. Datasets were exported in DICOM format.

### Measurements

**Fig 2:**
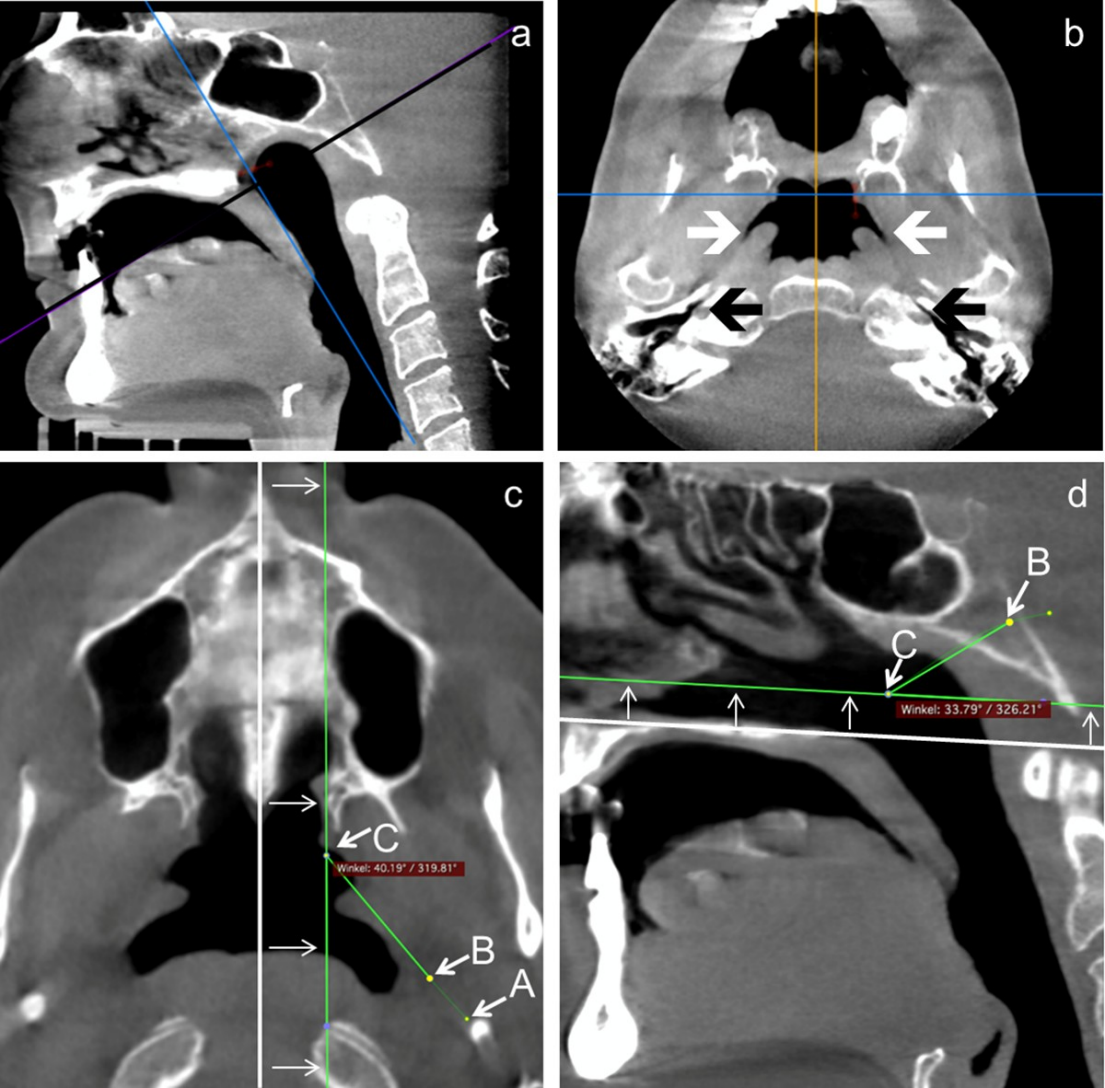
Screenshots of the scans during evaluation in Osirix. **a**: Sagittal viewing plane of one of the patients. The black line indicates the viewing plane in B. **b**: Virtual plane through the head with pharyngeal orifices (marked by white arrows) and tympanic orifices (black arrows). **c**: Determination of the angle between the cartilaginous ET and the sagittal plane. Points A, B, C indicate the tympanic orifice, the transition between bony and cartilaginous parts of the ET and the pharyngeal orifice, respectively. The sagittal plane (white line) was defined by the nasal spines and then moved aside until point C was reached. The angle between the connection B-C and the sagittal plane was measured. Point A is indicated but actually not in the viewing plane. **d**: Determination of the angle between the cartilaginous ET (connection B–C) and the horizontal plane. The horizontal plane (white line) was determined by the palatum durum and then moved until point C was met.

**Fig 3:**
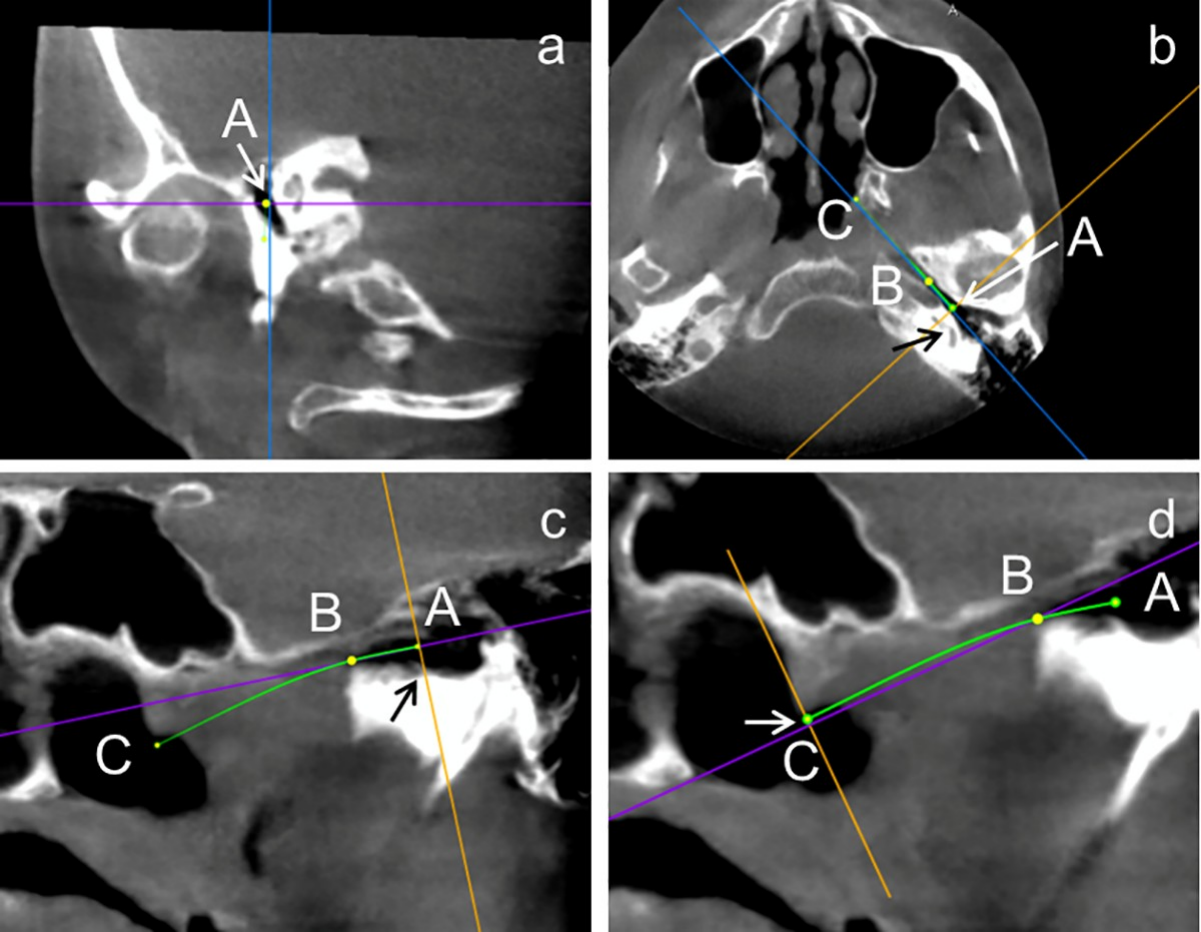
Screenshots of the detailed positioning of tympanic orifice and pharyngeal orifice. **a**: First rough position of point A, the marker of the TO. **b:** Moving point A to the center of the TO in the horizontal plane, where the basal turn of the cochlea is visible (indicated by the black arrow). **c:** The visual plane was turned to have points A and B in the same plane. The line perpendicular to the connection of A and B was moved along the connection line until it met the transition from the bony tube to the hypotympanic recess (indicated by black arraw). Point A was then moved to the crossing of both lines. **d:** Same procedure as in c but for the pharyngeal orifice. Here, the medial end of the torus tubarius was taken as reference for the auxiliary line perpendicular to the connection B to C (white arrow).

Datasets were imported in Osirix MD V1.0.2.16 (pixmeo SARL, Bernex, Switzerland), anonymized and used for virtual 3D reconstruction of the images and presented in CT-bone mode with only minimal adjustments regarding contrast and brightness. These parameters remained constant for all measurements, if not otherwise stated (Fig 2A and 2B). Measurements were performed using the 3D Curved MPR tool. In a first step, points A (tympanic orifice of the ET) (Fig 3A), B (transition from cartilaginous to bony ET), and C (pharyngeal orifice of the ET) were roughly defined (Curved Path tool) to determine the course of the ET. In the horizontal plane where the basal turn of the cochlea is located regularly at the same plane as the tympanic orifice (TO) of the bony ET, point A was moved to the center of the orifice (Fig 3B). In the same way also positions of points B (isthmus) and C (pharyngeal orifice, PO) were corrected such that both were positioned in the center of the ET. After turning the virtual image in a way that points A and B were visible in the same plane, positions of both were corrected in order to have point A directly at the intersection from the hypotympanic recess to the bony part of the tube (Fig 3C). Point B was set at the first opening of the circular bone of the bony ET. This point served as marker for the transition from the bony to the cartilaginous ET. With a further turning of the viewing planes points B and C became visible. Now, position of point C was corrected such that it was moved to be just beneath the lateral wall of the torus tubarius and the connection of both points was mainly within the tube (as far as it was visually detectable) (Fig 3D). The definition of these 3 points provided the basis for all further measurements.

The distances A-B (bony ET) and B-C (cartilaginous ET) were determined and the total length was calculated by summation of both. Additionally, the direct distance A-C was measured. To determine how far the ET appears open in the scans in the naturally closed condition, the viewing plane was set to be perpendicular to the axis of the cartilaginous ET and moved from point C to the end of the air filled part of the ET as the connection line B-C must not necessarily go through this point. The referring distance could then be measured in a perpendicular plane to the described one.

The diameter of the PO was measured when moving the viewing plane along the B-C axis to the point where the cartilage is closed around the air filled lumen of the tube for the first time when seen from nasopharynx. The largest diameter and the diameter perpendicular to the largest one were measured. Additionally, the distance of this point from C was determined. To further enhance contrast, these measurements were performed with standard WW/WL and CLUT/Perfusion settings.

To determine the diameter of the bony ET at the TO, CT-Bone and CLUT/Perfusion settings were applied. Largest diameter and the diameter perpendicular to it were measured at point A when the viewing plane was perpendicular to the axis A-B. Half way between A and B the diameter of the bony ET was measured again as described above.

For designing instruments for safe insertion of stents in the ET it is also important to know the distance / space between nasal conchae and pharyngeal orifice of the ET. Starting from point C (PO) an additional point was defined marking the posterior end of the concha nasalis inferior. The distance between both points was then determined as measure for the distance between the nasal conchae and the PO.

To determine the position of the axis of the ET in the head, angles in relation to horizontal and sagittal planes were determined. The sagittal plane was defined by anterior and posterior nasal spines. This plane was then moved aside to point C (Fig 2C). The horizontal plane was defined by the palatum durum. Also this plane was moved parallel to point C (Fig 2D). The angles of the projections of B-C and A-C on the sagittal plane were measured against the horizontal plane having C in the angular point. Same procedure was repeated for the projections of B-C and A-C on the horizontal plane and angles were measured against the sagittal plane. From these measured angles, effective curvature of the tube and the actual deviation of the axes of the ET from sagittal and horizontal planes could be calculated. Additionally, the deviation of the axis of the cartilaginous part of the ET from the insertion direction of any instrument through the nose parallel to the intersection of horizontal and sagittal planes was calculated.

### Statistical evaluation

Statistical evaluation was performed using Prism version 7.04 (GraphPad, LaJolla, US). Patients were divided into 9 groups according to age as follows: G0: <10, G1: 10–19, G2: 20–29, G3: 30–39, G4: 40–49, G5: 50–59, G6: 60–69, G7: 70–79, and G8: >79 years of age. Additionally young children (4–7 years) were compared with older patients (>7). Male and female patients just as left and right ET’s were evaluated separately. Data were first tested for Gaussian distribution using D’Agostino Pearson omnibus test. If data followed a Gaussian distribution, ANOVA followed by Tukey post-hoc tests or paired (left-right comparison) or unpaired (male-female) t-tests were applied. Otherwise, Wilcoxon matched pairs signed rank tests, Mann-Whitney tests (unpaired) or Kruskal-Wallis tests (more than 2 groups) followed by Dunn’s post hoc test were used.

## Results

A summary of all measures is given in Table 1. In the following the focus is set on the ones of larger importance for a possible stent application. Data for patients with different pre-existing conditions such as cleft palate are presented in Table 2.

**Table 1:** Summary of the measured and calculated distances (in mm) and angles (in °).

Parameter	All patients (n = 254)	Left side (n = 127)	Right side (n = 127)	Male (n = 138)	Female (n = 116)
Length bony ET [mm]	11.7 ± 1.8	11.5 ± 1.7	11.9 ± 1.9*	12.1 ± 1.7	11.2 ± 1.7***
Length cartilaginous ET [mm]	28.6 ± 2.5	28.5 ± 2.5	28.6 ± 2.5	29.2 ± 2.4	27.8 ± 2.4***
Total length [mm]	40.3 ± 2.6	40.0 ± 2.6	40.5 ± 2.6	41.3 ± 2.6	39.0 ± 2.1***
Length of air filled ET from PO [mm]	8.5 ± 3.8	8.2 ± 3.6	8.8 ± 3.9	7.6 ± 3.3	9.6 ± 4.0***
Distance PO to concha nasalis inferior [mm]	7.8 ± 1.9	7.8 ± 2.0	7.8 ± 1.9	7.8 ± 1.8	7.8 ± 2.1
Angle between axis cart. ET and sagittal plane [°]	41.3 ± 4.7	41.3 ± 4.8	41.3 ± 4.6	41.2 ± 4.1	41.5 ± 5.3
Angle between axis cart. ET and horizontal plane [°]	34.5 ± 6.8	35.4 ± 6.9	33.7 ± 6.7**	34.9 ± 6.4	34.1 ± 7.3
Angle of ET (curvature of ET) [°]	165.1 ± 6.3	165.5 ± 6.4	164.7 ± 6.3	165.2 ± 5.7	165.1 ± 7.0
Largest diameter at PO [mm]	9.2 ± 2.3	9.1 ± 2.4	9.2 ± 2.3	9.5 ± 2.5	8.7 ± 2.1**
… and at 90° [mm]	3.4 ± 1.2	3.5 ± 1.2	3.4 ± 1.2	3.4 ± 1.1	3.5 ± 1.2
Distance of measurement from PO [mm]	2.0 ± 1.2	1.8 ± 1.1	2.2 ± 1.2*	1.4 ± 1.0	2.2 ± 1.3***
Maximal width of torus tubarius [mm]	7.2 ± 1.1	7.0 ± 1.0	7.3 ± 1.2	7.3 ± 1.2	7.0 ± 1.0
Largest diameter at TO [mm]	6.8 ± 2.4	6.8 ± 2.5	6.9 ± 2.4	6.5 ± 2.2	7.1 ± 2.6
… and at 90° [mm]	3.0 ± 0.8	2.7 ± 0.7	3.2 ± 0.8	3.1 ± 0.8	2.8 ± 0.7
Largest diameter half way between A and B [mm]	4.3 ± 1.9	4.1 ± 2.0	4.5 ± 1.8	4.0 ± 1.9	4.6 ± 1.8
.. and at 90° [mm]	2.2 ± 0.9	2.0 ± 1.0	2.3 ± 0.9	2.1 ± 1.0	2.3 ± 0.9
Angle between axis bony ET and sagittal plane [°]	43.3 ± 4.0	43.2 ± 4.4	43.4 ± 3.5	43.1 ± 3.7	43.6 ± 4.3
Angle between axis bony ET and horizontal plane [°]	30.2 ± 5.8	31.0 ± 5.9	29.3 ± 5.6	30.2 ± 5.7	30.1 ± 5.9
Angle between insertion axis of tool and cart. ET [°]	53.6 ± 3.3	53.8 ± 3.3	53.4 ± 3.3	53.6 ± 2.9	53.6 ± 3.7

Significant differences between sides are indicated behind values for the right side. Significant differences between genders are indicated behind values for females.

PO–pharyngeal orifice; TO–tympanic orifice; cart.–cartilaginous

* p < 0.05

** p < 0.01

*** p < 0.001; n–number of ears included

**Table 2:** Overview on values for patients with reported pre-existing conditions or interventions as far as these could be measured.

	Cleft palate (n = 6)	Dysgnathia (n = 14)	Anomaly (n = 6)	Tumor (n = 2)
Length bony ET [mm]	10.0 ± 1.6	11.2 ± 1.7	13.4 ± 1.6	12.3 ± 1.0
Length cartilaginous ET [mm]	26.4 ± 2.7	28.3 ± 3.7	27.2 ± 2.1	27.3 ± 1.2
Total length [mm]	36.4 ± 2.0	39.4 ± 3.7	40.5 ± 3.0	39.6 ± 0.2
Length of air filled ET from PO [mm]	8.1 ± 0.8	7.5 ± 3.0	7.9 ± 2.2	6.2 ± 1.7
Distance PO to concha nasalis inferior [mm]	8.7 ± 0.9	7.9 ± 1.3	7.3 ± 2.2	7.7 ± 1.8
Angle between axis cart. ET and sagittal plane [°]	41.2 ± 2.9	42.2 ± 6.5	42.5 ± 1.2	42.8 ± 1.7
Angle between axis cart. ET and horizontal plane [°]	22.5 ± 7.4	34.5 ± 14.9	27.0 ± 8.5	31.1 ± 3.4
Angle of ET (curvature of ET) [°]	169.0 ± 6.5	162.5 ± 5.6	164.8 ± 7.0	170.7 ± 4.7
Largest diameter at PO [mm]	9.1 ±1.9	8.5 ± 2.7	8.9 ± 2.4	n.m.
… and at 90° [mm]	2.3 ± 0.3	2.8 ± 0.6	3.0 ± 0.6	n.m.
Distance of measurement from PO [mm]	0.8 ± 0.6	1.4 ± 1.4	2.7 ± 1.9	n.m.
Maximal width of torus tubarius [mm]	6.0 ± 0.9	7.2 ± 1.4	7.3 ± 0.9	4.8 ± 0.3
Largest diameter at TO [mm]	6.7 ± 1.5	7.0 ± 2.1	9.4 ± 2.8	n.m.
… and at 90° [mm]	3.0 ± 0.5	3.1 ± 0.9	3.3 ± 0.7	4.4
Largest diameter half way between A and B [mm]	3.7 ± 1.0	3.9 ± 1.5	5.7 ± 2.8	2.8
.. and at 90° [mm]	1.9 ± 0.7	1.9 ± 1.0	2.6 ± 1.4	n.m.
Angle between axis bony ET and sagittal plane [°]	42.7 ± 3.1	44.2 ± 4.7	44.7 ± 2.3	43.3 ± 0.5
Angle between axis bony ET and horizontal plane [°]	18.9 ± 6.1	29.9 ± 13.7	22.7 ± 6.7	28.4 ± 1.2
Angle between insertion axis of tool and cart. ET [°]	50.8 ± 2.7	55.2 ± 3.2	50.7 ± 1.6	51.2 ± 0.4

PO–pharyngeal orifice; TO–tympanic orifice; cart.–cartilaginous; n.m.–not measurable; n–number of ears included

**Fig 4:**
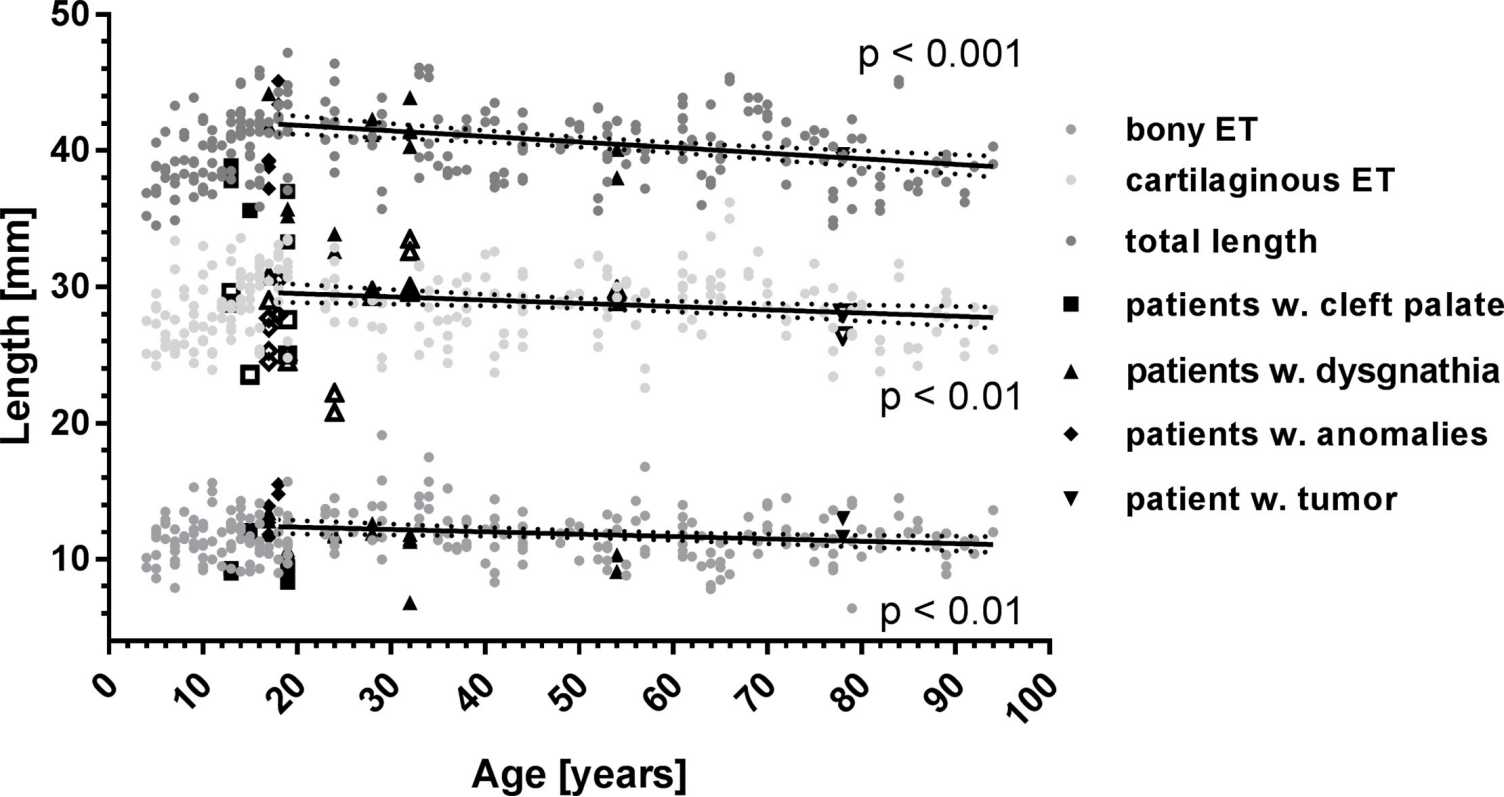
Lengths of cartilaginous and bony parts of the ET of all patients together with the total length of the ET. Additionally indicated are values for patients with pre-existing conditions such as cleft palate (□), dysgnathia (△), anomalies (◇), or a tumor (▽). Open symbols refer to cartilaginous parts whereas filled symbols belong to bony parts and the entire ET. Also provided are best linear fits for all adult patients with normal conditions, their 95% confidence intervals and p-values. The slopes of the linear fits are different from zero for all compartments indicating a reduction in length with higher age.

**Fig 5:**
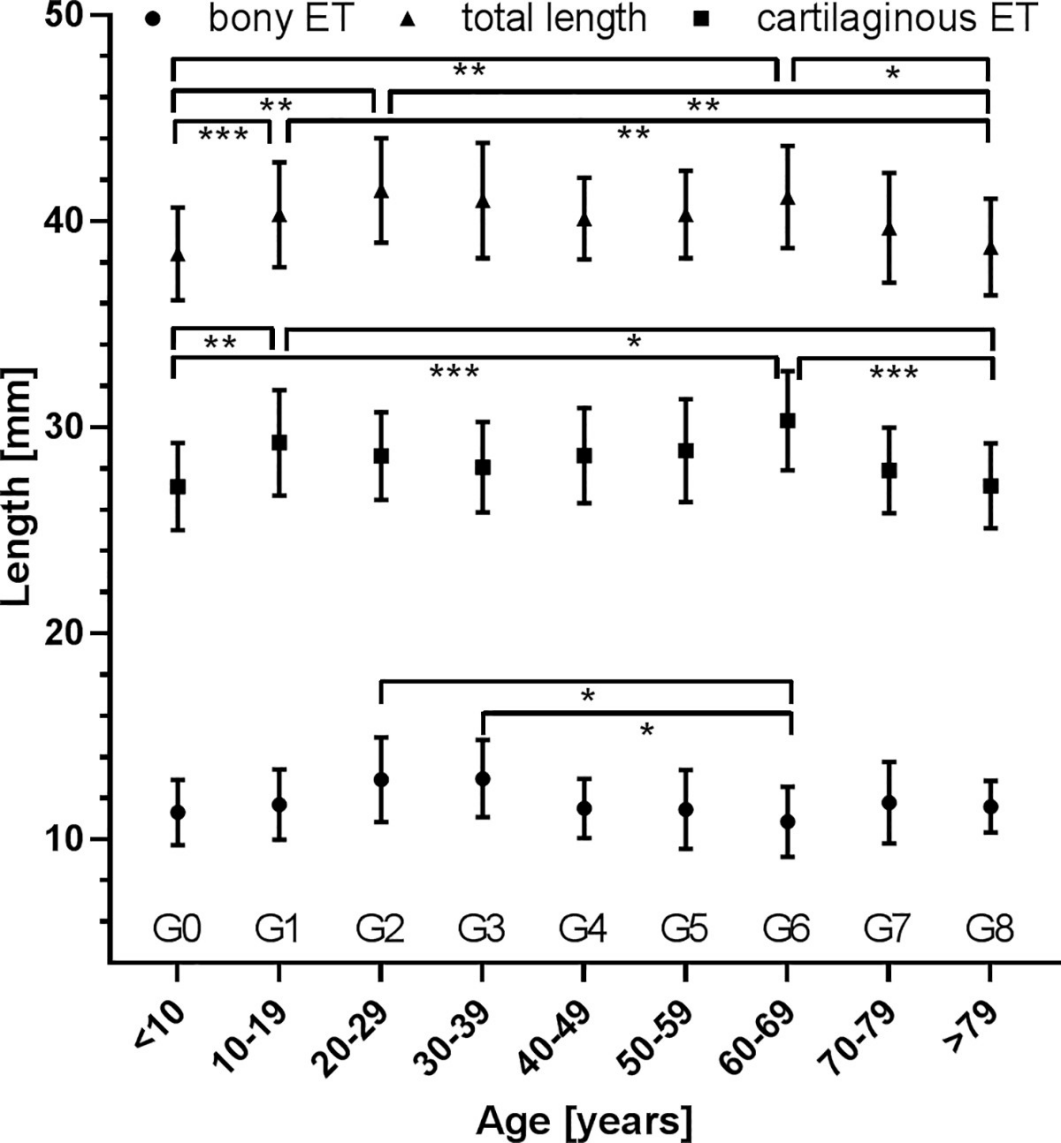
Total length of the ET (▲) and lengths of cartilaginous (■) and bony parts (●) of all patients without pre-existing conditions as sorted according to the age groups. Presented are mean values ± standard deviations (SD). * p < 0.05; ** p < 0.01; *** p < 0.001.

The length of the ET was measured to be 40.3 mm ± 2.6 mm with a minimum of 34.5 mm and a maximum of 47.2 mm. The cartilaginous part comprised 28.6 mm ± 2.5 mm (min: 22.6 mm, max: 36.2 mm) and the bony part 11.7 mm ± 1.8 mm (min: 6.4 mm, max: 19.1 mm). The male ET was significantly (p < 0.001) longer than the female ET. Average values for females and males as well as left and right are provided in Table 1. For younger children (age 4–7) the ET was shorter (length: 37.9 mm; p < 0.001) than in older patients (length: 40.1 mm). Similarly, differences were found when comparing age group G0 to groups G1, G2, G6, but also for group G8 in comparison to groups G1, G2, and G6 with G0 and G8 being shorter than the other groups. Age dependent comparison of the lengths of the cartilaginous ET resulted in differences between group G0 and groups G1 and G6, and between group G8 and groups G1 and G6. In these comparisons always the first mentioned groups had shorter cartilaginous ETs. The length of the bony ET was shorter in group G6 compared to groups G2 and G3. Individual data for the lengths of bony and cartilaginous parts and the entire ET are presented in Fig 4. Group mean values and standard deviations are additionally presented in Fig 5.

**Fig 6:**
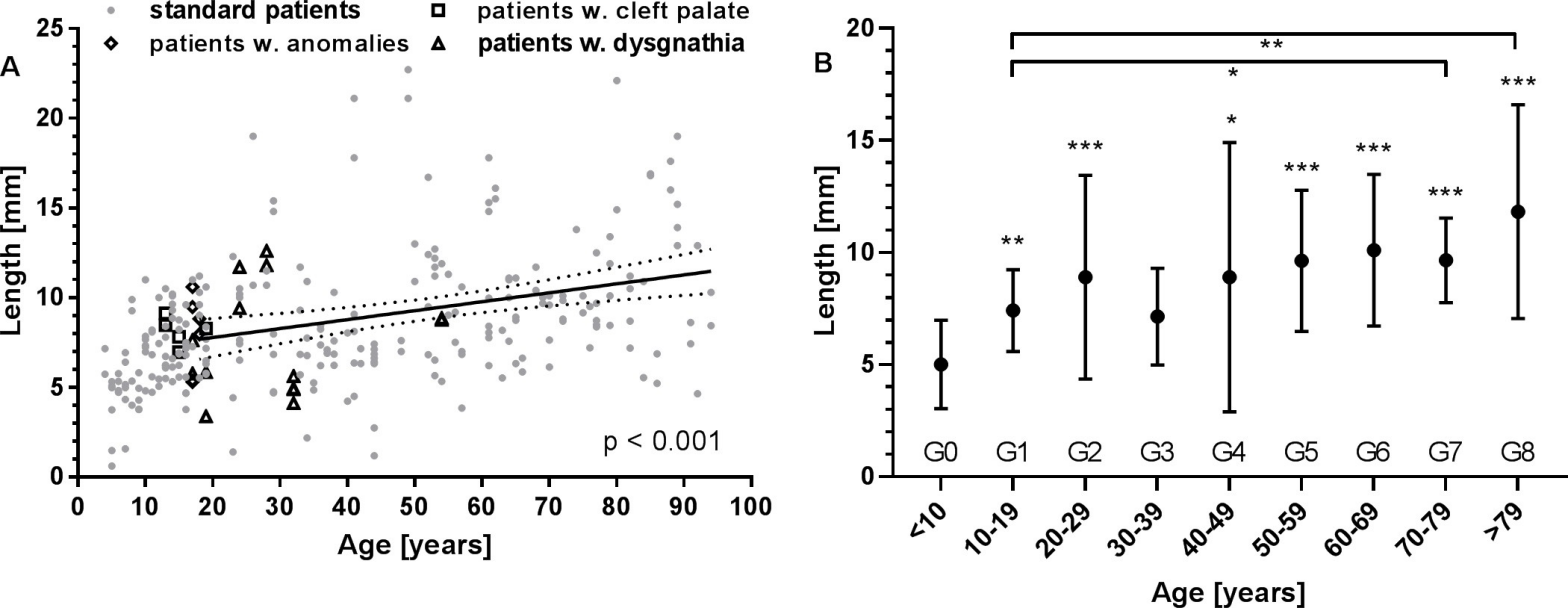
Lengths of the naturally open (air filled) part of the cartilaginous ET. An increase with age can be detected. **A:** Individual data for all patients. Additionally indicated are values for patients with pre-existing conditions such as cleft palate (□), dysgnathia (△), and anomalies (◇). Best linear fit for all adult patients with normal conditions, its 95% confidence intervals and p-value are provided. **B:** Group mean values for all patients without pre-existing conditions (mean ± SD). * p < 0.05; ** p < 0.01; *** p < 0.001.

The detected opening of the ET from the pharyngeal orifice was on average 8.5 mm ± 3.8 mm (Fig 6A) but was significantly (p < 0.001) shorter in males (7.6 mm) than in females (9.6 mm). This air filled part of the ET increased with age from 5.0 mm ± 2 mm (group G0) via 7.4 mm ± 1.8 mm (G1), 8.9 mm ± 4.5 mm (G2) to 11.8 mm ± 4.8 mm for the oldest patients (group G8; Fig 6B) and was significantly smaller in patients aged 4–7 compared to older ones (p < 0.001).

The largest diameter of the cartilaginous ET as measured close to the PO was smaller (p < 0.01) in females than in males (compare Table 1) and for children at the age of 4–7 smaller than in older patients (p = 0.0002). No further age dependent differences were found. Findings were different for the diameter perpendicular to the largest one. Here, an increase with age was found, ranging from 2.5 mm ± 0.7 mm (group G0) to 4.3 mm ± 1.4 mm (group G8) with the differences between group G0 and groups G2 (p < 0.05), G5 (p < 0.05), G6 (p < 0.001), G7 (p < 0.001), G8 (p < 0.001), group G1 and groups G6 (p < 0.001), G7 (p < 0.001), G8 (p < 0.001), and group G7 and groups G3 (p < 0.05) and G4 (p < 0.05) being significant. The distance of the point of measurement (first circular closure of the cartilage around the still air filled lumen of the ET) from the PO was 2.0 mm ± 1.2 mm. This distance was larger for females than males (p < 0.001). Surprisingly, it was also smaller on the left side than on the right side (p < 0.001; all normal patients). No age dependent differences were found.

**Fig 7:**
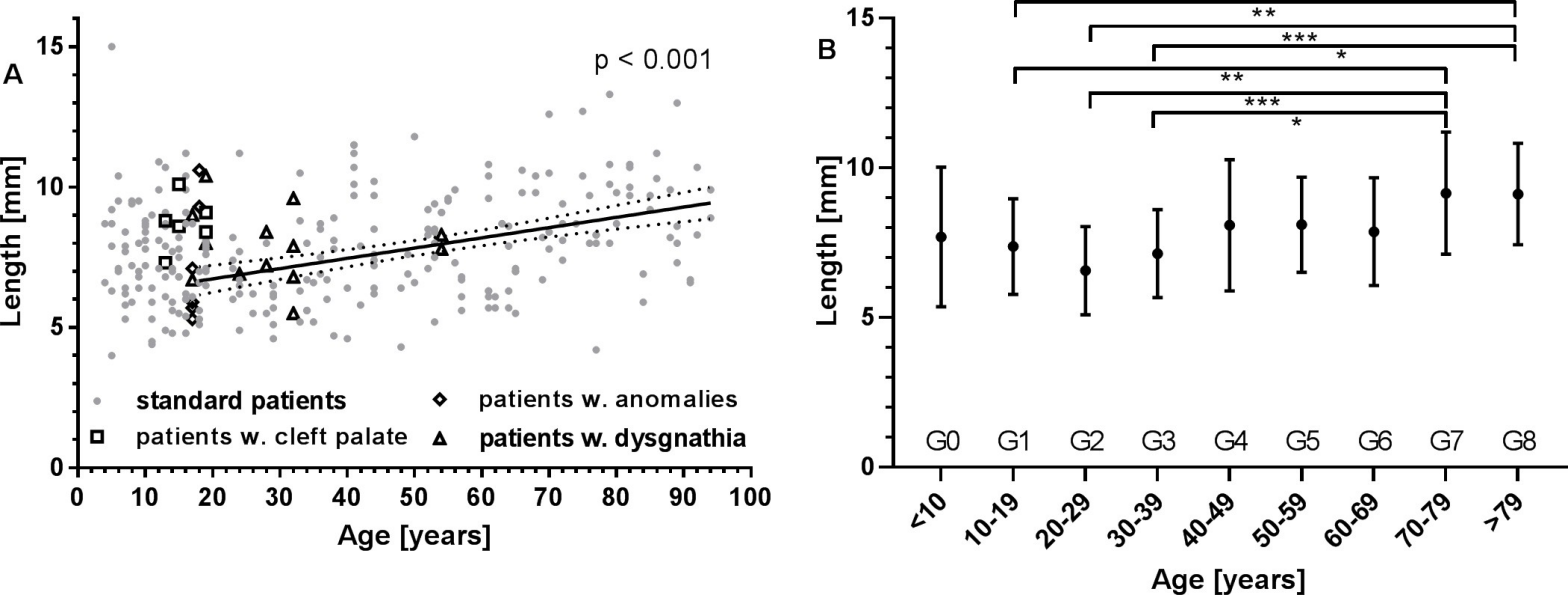
Distance of the pharyngeal orifice of the ET from the concha nasalis inferior. This distance is important for the development of tools for atraumatic stent insertion into the ET. **A:** Individual data for all patients. Additionally indicated are values for patients with pre-existing conditions such as cleft palate (□), dysgnathia (△), and anomalies (◇). Best linear fit for all adult patients with normal conditions, its 95% confidence intervals and p-value are provided. **B:** Group mean values for all patients without pre-existing conditions (mean ± SD). * p < 0.05; ** p < 0.01; *** p < 0.001.

The distance of the pharyngeal orifice from the concha nasalis inferior was 7.8 mm ± 1.9 mm (min: 4 mm, max: 15 mm). An age dependent increase was found between groups G1, G2, G3 on one side and G7 and G8 on the other side. An overview for all patients is provided in Fig 7.

**Fig 8:**
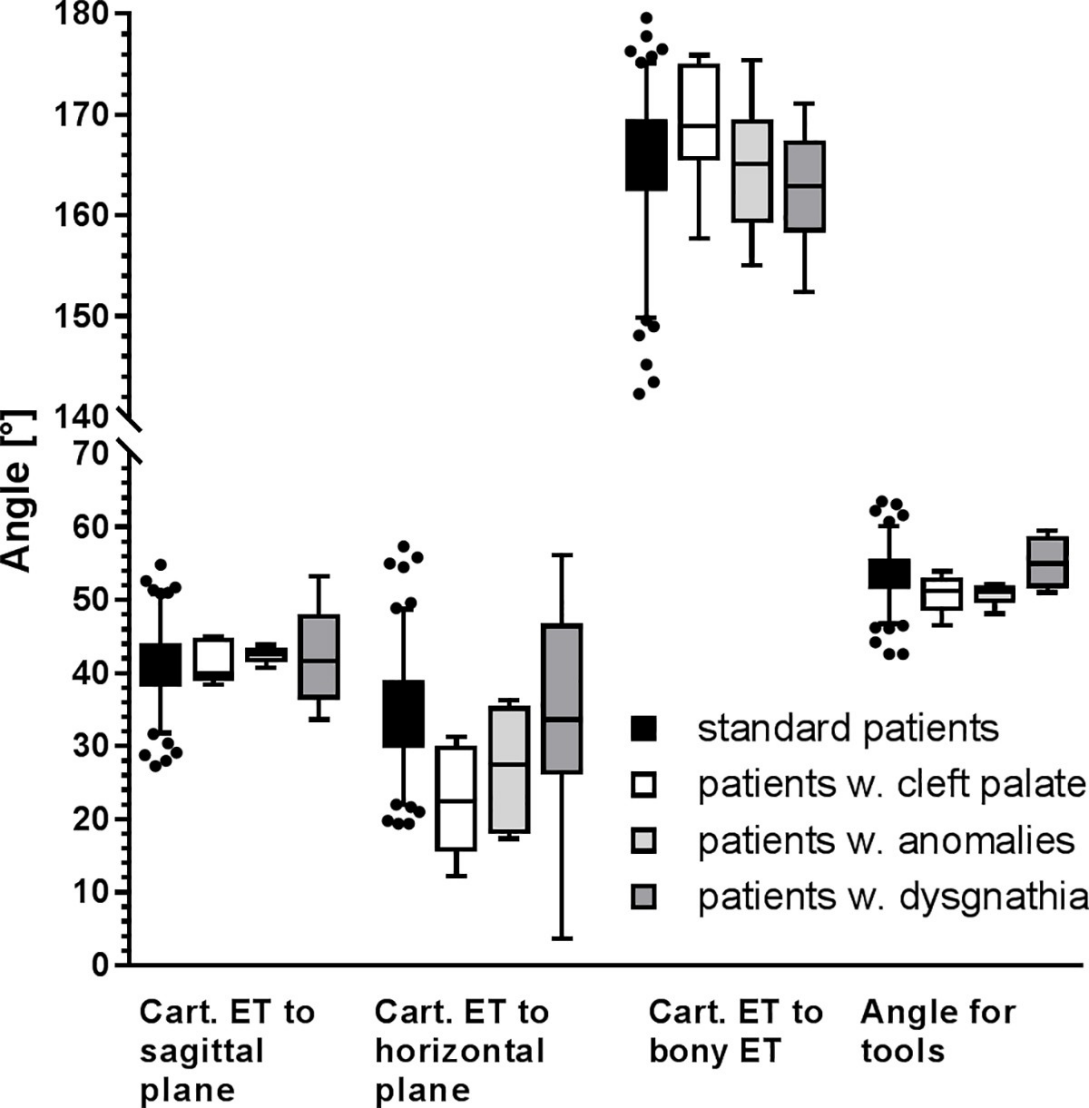
**Determined angles of the cartilaginous part of the ET with the sagittal (far left) and horizontal planes (central left) as well the angles between bony and cartilaginous parts (central right) and angles that should be covered by insertion tools (far right).** Upper and lower whiskers comprise 95% of values (2.5 to 97.5 percentile) and outliers are indicated. Values for standard patients are presented (black) as well as for cleft palate (white), anomalies (light grey) and dysgnathia (dark grey).

The angle between the axis of the cartilaginous ET (connection B-C) and the horizontal plane was 34.5° ± 6.8° (min: 19.4°, max: 57.3°) and was significantly smaller on the right side (p < 0.01). Only in age group G2 this angle was larger than in group G0 (p < 0.01). No differences were detected between genders (p = 0.2024) or between young children (4–7) and older patients (p = 0.1395). The angle between connection B-C and the sagittal plane was 41.3° ± 4.7° (min: 27.3°, max: 54.8°) with no significant differences at all. The curvature of the ET was calculated to be 165.1° ± 6.3° (min: 142.3°, max: 179.6°) (Fig 8) with no side, gender, or age specific differences.

Regarding the design of any application tools, the angle between the axis B-C and the insertion axis of an instrument through the nose parallel to horizontal and sagittal planes was calculated. On average the deviation from the insertion axis is 53.6° ± 3.3° (min: 42.6°; max: 63.5°). No differences were detected in any of the comparisons.

Patients with pre-existing conditions are included in Figs 4 and 6–8, but highlighted. Cleft palate patients might have slightly shorter cartilaginous ET’s and smaller angles in relation to the horizontal plane but due to the relatively low numbers, no statistical evaluation was performed.

## Discussion

In order to develop stents for application in the human ET it is crucial to have information on dimensions of the ET and its position in the head. Using a stent, ventilation of the middle ear via the ET shall be restored but a permanently open ET is to be avoided. To achieve this goal, the length of the stent will–if inserted only in the cartilaginous part of the ET–always be shorter than the length of the cartilaginous ET. Furthermore, in case the ET is naturally not completely closed all the way to the pharyngeal orifice, the possible length of the stent would get reduced further. In addition, it was known that the length of the ET is increasing with age in children [16], and a linear increase of the intranasal volume with age [17] was also described earlier. Therefore, age dependent changes in at least some measures being important for stent development were to be expected and in the current study, one focus was put on the evaluation of possible age-related changes.

CBCT images were used for this evaluation as images at high resolution are captured using less radiation compared to conventional CT scans [18,19]. Patients with any pre-existing conditions were excluded. For cleft palate patients it is known that an enlarged nasopharynx can be expected, leading to a compression of surrounding structures including the ET [20]. Patients with reported dysgnathia were excluded because the severity of dysgnathia could not be classified from the patient folders and at least for severe dysgnathia a possible influence on the anatomical structures of the pharynx was described earlier [21]. One included patient was found with a patulous ET that was not documented before. It cannot be ruled out whether this was an artifact due to yawning or swallowing during the scan. Nonetheless this seems to be very unlikely as movement artifacts would have been expected in the images as well.

Many different measures were collected and are just reported in Table 1. In results and discussion sections it was concentrated on the expectedly most important ones for the application of stents into the ET. The resolution of the scans was set to 0.3 mm. Furthermore, due to technical limitations and movement artifacts of patients, a resolution higher than 0.5 mm is not realistic [22]. This provides a relatively large error for all measurements (both ends of a line can be affected). Individual lengths can be under- or overestimated. Due to the relatively large number of patients an equal distribution can be assumed and average values are expected to be less affected. Additionally, as this is a retrospective study, scans of every patient were taken at optimal settings for the individual patient. Therefore, some differences in settings might have influenced the presentation of different tissues such as mucosal thickness.

Dimensions of the ET were reported earlier also by other groups. One more recent study [23] used CT scans and compared the lengths of the ET of children with and without otitis media with effusion with adults, but did not distinguish between cartilaginous and bony parts. They determined the total length of the tube of adults to be between 42.5 mm and 42.9 mm (left / right) respectively. The average length for all patients aged >7 in the current study was 40.5 mm. The decision to split the patient group at the age of 7 was done as it was earlier described that ET measures of approximately 7 years old children were already comparable to the ones of adults [23]. Moreover, using a single line from the pharyngeal orifice to the tympanic orifice as in Takasaki et al. [23], should lead to a slight underestimation due to missing the curvature of the ET. Therefore, the most likely explanation for the difference is the methodological difference in determination of especially the tympanic orifice. The values for children are perfectly comparable between both studies, but the average age in Takasaki et al. [23] was 4 years whereas it was 5.9 years in the current study. As the length of the ET is increasing with age in children [16], the measured length probably would have been smaller again in age matched children in the current study. It could also be that this difference is simply genetically determined due to the different origin of the two cohorts (Asian and Caucasian).

In older studies, the lengths of the entire ET were with 36.4 mm (range 34–40 mm) [24] and 36.2 mm (range 31.2–42.4 mm) [25] and the lengths of the cartilaginous part with 24.6 mm (range 21.5–27 mm) [24] and 25 mm (range 24–25 mm) [26,27] smaller than the 40.3 mm (entire ET) and the 28.6 mm for the cartilaginous part in the current study. However, these previous studies were performed in cadavers and due to fixation and histological evaluation little shrinkage was to be expected. Bone is less affected by shrinkage [28], indicating that the differences might be caused by shrinkage of the oft tissue in the old studies. Results for the length of the bony ET were with 13.4 mm [24], 7.6 mm [25] and 11–12 mm [26] better comparable to the 11.8 mm in the current study. Having found a range in length of the cartilaginous part of the ET from 22.6 mm to 36.2 mm suggests the requirement for an imaging of the ET before implantation of a stent to provide the best solution for the individual patient and maybe it also enables locating the position of the stenosis as suggested by Tarabichi and Kapadia [29].

Generally, the length of the ET was larger in males compared to females. This can most likely be explained by the different body size, even though not known for included patients. Furthermore, the right bony ET was significantly longer than the left bony ET even though the difference was with 0.5 mm very small. We can only speculate that this was found by chance due to the resolution of the images or that it is correlated with the described lower fossa cranii media on the right side [30].

With age, the length of the ET decreased, the open length of the cartilaginous part increased and the distance of the pharyngeal orifice from the conchae increased. This is especially true for patients being 70 years of age or older and can be explained by the clinical observation of an atrophic gland tissue in elderly people. An age related change in the epithelial layer of the ET was described earlier [31–33]. Additionally, a linear increase of the intranasal volume with age [17] and a decrease in gland tissue with age was also reported for the conchae nasalis [34]. As the distance between PO and concha nasalis inferior was measured, this could potentially also be influenced be any medications the patients used.

Of huge importance for an atraumatic insertion of stents into the ET are the angles against horizontal and sagittal planes. Due to the definition of the horizontal plane by the palatum durum and the sagittal plane by the nasal spines, their orientation was fixed. Therefore only the projections of the ET on these planes were measureable and the true angles had to be calculated. Our values (34.6°) are very similar to the values (34° - 36°) provided by literature [5,34], when also the palatum durum was taken as reference and also comparable to the 35.6° as given by Pahnke [25] and measured against the Frankfort horizontal plane. Values given by Takasaki et al. [23] were with 27.3° smaller and measured against Reid’s baseline. If the 7° difference between Reid’s baseline and the plane of the palatum durum is taken into account, also this value fits very well. Same is true for the angle against the sagittal plane, were 42° (range 33–50°) are reported for measurements at cadavers [35]. The deviation of the course of the cartilaginous part of the ET from the insertion axis of any tool (parallel to the intersection of horizontal and sagittal plane) was then calculated to be 53.7° on average with a range from 42.6° to 63.5°. This means, any tools to manipulate the ET or to insert stents into the ET as atraumatic as possible should cover angles from at least 42° to 64°, even better add some more variability to really fit all patients.

In the current study the angle between cartilaginous ET and horizontal plane was with a difference of 1.7° significantly smaller on the right side compared to the left one. A difference between sides of 3° was already described earlier by Pahnke and can be explained by the above mentioned lower Fossa cranii media on the right side [30].

It is known that the slope of the ET is shallower in young children [16]. A difference between children and adults was not found within the current study. This could have been missed in our data because only 4 children were 4 or 5 years old. For children aged between 1500 and 2000 days (approximately 5 years), angles are to be expected in the angle range found in adults [23].

## Conclusion

The current study provides the most detailed collection of different measures regarding dimensions and position of the ET in humans available so far. Additionally, it provides specifications that stents and tools for positioning of a stent should cover.

## References

[1] Rovers MM. The burden of otitis media. Vaccine. 2008;26 Suppl 7: G2–4. 19094933 10.1016/j.vaccine.2008.11.005

[2] Schroder S, Ebmeyer J. [Diagnosis and treatment of Eustachian tube dysfunction]. HNO. 2018;66: 155–166. 10.1007/s00106-017-0465-2 29313115

[3] Pau HW. Eustachian tube and middle ear mechanics. HNO. 2011;59: 953–963. 10.1007/s00106-011-2368-y 21909770

[4] Steinbach E, Pusalkar A, Heumann H. Cholesteatoma—pathology and treatment. Adv Otorhinolaryngol 1988;39: 94–106. 10.1159/000415658 3394569

[5] Smith ME, Scoffings DJ, Tysome JR. Imaging of the Eustachian tube and its function: a systematic review. Neuroradiology. 2016;58: 543–556. 10.1007/s00234-016-1663-4 26922743 PMC4877436

[6] Alper CM, Swarts JD, Singla A, Banks J, Doyle WJ. Relationship between the electromyographic activity of the paratubal muscles and eustachian tube opening assessed by sonotubometry and videoendoscopy. Arch Otolaryngol Head Neck Surg. 2012;138: 741–746. 10.1001/archoto.2012.1293 22801708

[7] Sudhoff HH, Mueller S. Treatment of pharyngotympanic tube dysfunction. Auris Nasus Larynx. 2018;45: 207–214. 10.1016/j.anl.2017.07.001 28734727

[8] Browning GG, Gatehouse S. The prevalence of middle ear disease in the adult British population. ClinOtolaryngol. 1992;17: 317–321. 10.1111/j.1365-2273.1992.tb01004.x 1526050

[9] Norman G, Llewellyn A, Harden M, Coatesworth A, Kimberling D, Schilder A, et al Systematic review of the limited evidence base for treatments of Eustachian tube dysfunction: a health technology assessment. Clinical otolaryngology. 2014;39: 6–21. 10.1111/coa.12220 24438176

[10] Kujawski OB, Poe DS. Laser eustachian tuboplasty. Otol Neurotol. 2004;25: 1–8. 10.1097/00129492-200401000-00001 14724483

[11] Ockermann T, Reineke U, Upile T, Ebmeyer J, Sudhoff HH. Balloon dilatation eustachian tuboplasty: a clinical study. Laryngoscope. 2010;120: 1411–1416. 10.1002/lary.20950 20564474

[12] Silvola J, Kivekas I, Poe DS. Balloon Dilation of the Cartilaginous Portion of the Eustachian Tube. Otolaryngol Head Neck Surg. 2014;151: 125–130. 10.1177/0194599814529538 24705223

[13] Llewellyn A, Norman G, Harden M, Coatesworth A, Kimberling D, Schilder A, et al Interventions for adult Eustachian tube dysfunction: a systematic review. Health Technol Assess. 2014;18: 1–180, v-vi. 10.3310/hta18460 PMC4781384 25029951

[14] Miller F, Burghard A, Salcher R, Scheper V, Leibold W, Lenarz T, et al Treatment of middle ear ventilation disorders: sheep as animal model for stenting the human Eustachian tube—a cadaver study. PloS one. 2014;9: e113906 10.1371/journal.pone.0113906 25419714 PMC4242708

[15] Pohl F, Schuon RA, Miller F, Kampmann A, Bultmann E, Hartmann C, et al Stenting the Eustachian tube to treat chronic otitis media—a feasibility study in sheep. Head & face medicine. 2018;14(1): 8 10.1186/s13005-018-0165-5 29728102 PMC5935938

[16] Lang J. Klinische Anatomie des Ohres. Wien, Springer Verlag, 1992.

[17] Loftus PA, Wise SK, Nieto D, Panella N, Aiken A, DelGaudio JM. Intranasal volume increases with age: Computed tomography volumetric analysis in adults. Laryngoscope. 2016;126: 2212–2215. 10.1002/lary.26064 27261245

[18] Chau AC, Fung K. Comparison of radiation dose for implant imaging using conventional spiral tomography, computed tomography, and cone-beam computed tomography. Oral surgery, oral medicine, oral pathology, oral radiology, and endodontics. 2009;107: 559–565. 10.1016/j.tripleo.2008.11.009 19168378

[19] Loubele M, Bogaerts R, Van Dijck E, Pauwels R, Vanheusden S, Suetens P, et al Comparison between effective radiation dose of CBCT and MSCT scanners for dentomaxillofacial applications. European journal of radiology. 2009;71: 461–468. 10.1016/j.ejrad.2008.06.002 18639404

[20] Rajion ZA, Al-Khatib AR, Netherway DJ, Townsend GC, Anderson PJ, McLean NR, et al The nasopharynx in infants with cleft lip and palate. Int J Pediatr Otorhinolaryngol. 2012;76: 227–234. 10.1016/j.ijporl.2011.11.008 22136741

[21] Al-Moraissi EA, Al-Magaleh SM, Iskandar RA, Al-Hendi EA. Impact on the pharyngeal airway space of different orthognathic procedures for the prognathic mandible. Int J Oral Maxillofac Surg. 2015;44(9): 1110–1118. 10.1016/j.ijom.2015.05.006 26025815

[22] Brüllmann D, Schulze RK. Spatial resolution in CBCT machines for dental/maxillofacial applications-what do we know today? Dentomaxillofac Radiol. 2015;44(1):20140204 10.1259/dmfr.20140204 25168812 PMC4614158

[23] Takasaki K, Takahashi H, Miyamoto I, Yoshida H, Yamamoto-Fukuda T, Enatsu K, et al Measurement of angle and length of the eustachian tube on computed tomography using the multiplanar reconstruction technique. Laryngoscope. 2007;117: 1251–1254. 10.1097/MLG.0b013e318058a09f 17603324

[24] Bezold F. Die Corrosions-Anatomie des Ohres. München: Literarisch-artistische anstalt (T. Riedel); 1882 24–31.

[25] Pahnke J. Morphologie, Funktion und Klinik der Tuba Eustachii. Laryngo-Rhino-Otol. 2000;79: S1–S21.

[26] Proctor B. Embryology and anatomy of the eustachian tube. Arch Otolaryngol. 1967;86: 503–514. 10.1001/archotol.1967.00760050505008 4952840

[27] Proctor B. Anatomy of the eustachian tube. Arch Otolaryngol. 1973;97: 2–8. 10.1001/archotol.1973.00780010006002 4684903

[28] Buytaert J, Goyens J, De Greef D, Aerts P, Dirckx J. Volume shrinkage of bone, brain and muscle tissue in sample preparation for micro-CT and light sheet fluorescence microscopy (LSFM). Microscopy and microanalysis. 2014;20: 1208–1217. 10.1017/S1431927614001329 24963987

[29] Tarabichi M, Kapadia M. Preoperative and Intraoperative Evaluation of the Eustachian Tube in Chronic Ear Surgery. Otolaryngol Clin North Am. 2016;49: 1135–1147. 10.1016/j.otc.2016.05.004 27468635

[30] Lang J. Neuroanatomie der Nn. opticus, trigeminus, facialis, glossopharyngeus, vagus, accessorius und hypoglossus. Arch Otorhinolaryngol 1981;231: 1–69. 10.1007/bf00465556 7020666

[31] Renteria AE, Mfuna Endam L, Desrosiers M. Do Aging Factors Influence the Clinical Presentation and Management of Chronic Rhinosinusitis? Otolaryngol Head Neck Surg. 2017;156: 598–605. 10.1177/0194599817691258 28195747

[32] Schrodter S, Biermann E, Halata Z. Histological evaluation of age-related changes in human respiratory mucosa of the middle turbinate. Anatomy and embryology. 2003;207: 19–27. 10.1007/s00429-003-0326-5 12783321

[33] Tomoda K, Morii S, Yamashita T, Kumazawa T. Deviation with increasing age in histologic appearance of submucosal glands in human eustachian tubes. Acta Otolaryngol. 1981;92: 463–467. 10.3109/00016488109133285 7315265

[34] Berger G, Balum-Azim M, Ophir D. The normal inferior turbinate: histomorphometric analysis and clinical implications. Laryngoscope. 2003;113: 1192–1198. 10.1097/00005537-200307000-00015 12838018

[35] Prades JM, Dumollard JM, Calloc'h F, Merzougui N, Veyret C, Martin C. Descriptive anatomy of the human auditory tube. Surgical and radiologic anatomy. 1998;20: 335–340. 10.1007/bf01630616 9894313

